# Risperidone-Associated Neuroleptic Malignant Syndrome in an Inpatient With Schizophrenia, With Successful Rechallenge and 3 Year Follow-Up

**DOI:** 10.3389/fpsyt.2018.00718

**Published:** 2018-12-18

**Authors:** Ting-Ren Chen, Ying-Chang Chen

**Affiliations:** ^1^Department of Psychiatry, Chang Bing Show Chwan Memorial Hospital, Lukang, Taiwan; ^2^Institute of Hospital and Health Care Administration, National Yang-Ming University, Taipei, Taiwan; ^3^Department of Psychiatry, Show Chwan Memorial Hospital, Changhua, Taiwan

**Keywords:** neuroleptic malignant syndrome, antipsychotics, risperidone, schizophrenia, hyperthermia

## Abstract

Neuroleptic malignant syndrome (NMS) is rare but one of the most serious adverse effects of antipsychotics. Here, we report a case of risperidone-associated NMS in which a successful rechallenge of risperidone was observed with a positive follow-up. A 47-year-old female with schizophrenia was treated with risperidone 4 mg/d for 8 months in 2009 and was admitted to our hospital in 2015 owing to violent behavior under persecutory delusions. Risperidone 2 mg/d was initiated and increased to 4 mg/d 54 days later. Further, long-acting injectable (LAI) risperidone 25 mg per 2 weeks was added on hospital day 15. On hospital day 116, NMS occurred and thus we discontinued all antipsychotics including LAI risperidone, then NMS improved. We resumed LAI risperidone 25 mg per 2 weeks on hospital day 148, thus we waited for 22 days before re-starting the drug treatment. She was discharged on hospital day 371, then switched to LAI paliperidone 150 mg per 4 weeks 2 months later. At the time of a follow-up 3 years later, NMS had not reoccurred. This case reports on an unusual presentation of NMS in which no hyperthermia was observed. Furthermore, this case indicated that NMS may occur in a dose-dependent manner. In conclusion, this case reported important information for clinicians with regard to antipsychotic drug rechallenges and proper dosing of APs to avoid or reverse NMS.

## Introduction

A 47-year-old female with schizophrenia and without other neuropsychiatric or systemic illnesses was treated with risperidone 4 mg/d for 8 months in 2009. In 2015, she was admitted owing to the violent behavior of attacking her mother-in-law under persecutory delusion with the belief that her mother-in-law was going to murder her and auditory hallucination of hearing her mother-in-law criticize her behind her back. Risperidone 2 mg/d was initiated and increased to 4 mg/d 54 days later. Further, long-acting injectable (LAI) risperidone 25 mg per 2 weeks was added on hospital day 15. She did not receive any mood stabilizers on admission, such as lithium, carbamazepine, valproate. During treatment, the patient complained of soreness and weakness of her whole body, and refused to eat or ambulate on hospital day 116, at which point she was tachycardic with a bpm of 116, but afebrile (36.4°C) with stable blood pressure (113/72 mm Hg). Physical examination revealed delirium in which her clinical manifestations were altered level of consciousness, disorganized speech and disorganized behavior of lying on the ground for no reasons; she also showed muscle rigidity and dysphagia. Laboratory studies were remarkable for alanine transaminase (ALT) of 659 U/L and aspartate transaminase (AST) of 182 U/L and elevated BUN/creatinine ratio of 16/1.27, as well as leukocytosis with left shift (WBC: 16,680/mm^3^; SEG: 80%). Urine examination showed occult blood (4+) without RBC and an urobilinogen level of 4. By hospital day 126, her serum creatine phosphokinase (CPK) was elevated to 3,196 U/L and all antipsychotics were immediately stopped. Urine exams, and ALT, and AST levels were normal 7 days after the discontinuation of all antipsychotics. Fourteen days following the discontinuation of all antipsychotics, her CPK level dropped to 282 U/L. Because the vital signs of patient were stable during the recovery from neuroleptic malignant syndrome (NMS), we did not apply dantrolene and bromocriptine, but the patient was given lorazepam 0.5 mg/d and the dose of biperiden was increased from 4 mg/d to 6 mg/d. IV hydration was also given. We resumed LAI risperidone 25 mg per 2 weeks on hospital day 148, thus we waited for 22 days before re-starting the drug treatment. She was discharged on hospital day 371, then switched to LAI paliperidone 150 mg per 4 weeks 2 months later. At the time of a follow-up 3 years later, NMS had not reoccurred.

## Background

NMS is rare but one of the most serious adverse effects of antipsychotics (APs) (1). The incidence rate of NMS ranges from < 0.1% to 1% and the mortality rate is approximately 10% (2, 3). NMS is an idiosyncratic reaction generally characterized by rigidity, tremor, hyperthermia, tachycardia, diaphoresis, dysphagia, labile blood pressure, dysregulated sympathetic nervous system hyperactivity, alterations in mental status ranging from confusion to coma, leukocytosis, low serum iron level and elevated creatine phosphokinase (CPK) levels (3–5). If NMS is not promptly diagnosed and treated, it can be fatal due to pneumonia, renal failure, cardiac arrest, pulmonary embolism, disseminated intravascular coagulation or permanent damages, such as neurological sequelae (6). NMS usually occurs within 1 month after initiating an antipsychotic treatment, but two-thirds of cases develop within the first week (1, 7). Not all atypical AP-induced NMS meet the criteria that include these symptoms (8–10), and drug rechallenge and follow-up care have not been highlighted in many reports of NMS (11). Here, we report a case of risperidone-associated NMS in which a successful rechallenge of risperidone was observed with a positive follow-up. Despite the fact that hyperthermia is considered a hallmark symptom in NMS, this patient's temperature was never >37°C throughout treatment.

## Discussion

This case demonstrates two interesting points worth discussing. First, no hyperthermia was observed. The patient had NMS symptoms including muscle rigidity, altered levels of consciousness, dysphagia, tachycardia, significant increases in CPK and transaminases, leukocytosis with left shift (3), and occult blood in urine without RBCs, possibly indicating myoglobinuria. Her symptoms decreased as the risperidone dose decreased, supporting the diagnosis of NMS. Generally, hyperthermia is the core NMS symptom, but this case demonstrates that without it, NMS can still occur (12). Second-generation antipsychotics (SGAs) such as clozapine, risperidone, and olanzapine have been found to be less likely to be associated with hyperthermia than first-generation antipsychotics. Some studies have shown that due to the partial agonistic action at 5-HT1A receptors by perospirone, clozapine, ziprasidone, quetiapine, and aripiprazole, it should be noted that when prescribing these SGAs especially combining with antidepressants, we need to carefully differentiate between atypical NMS and serotonin syndrome. Although it is difficult to differentiate between NMS and serotonin syndrome due to the overlapping symptoms, serotonin syndrome can be differentiated from NMS in part by physical examination. The clinical features associated with serotonin syndrome are clonus, hyperreflexia, tremor, mydriasis, and gastrointestinal syndromes, whereas severe muscle rigidity and bradykinesia are well-recognized clinical features of NMS (13). In our case, we did not combine another antipsychotics or antidepressants with risperidone. Also, risperidone is not the 5-HT1A receptor agonist (13), thus we can exclude the possibility of serotonin syndrome in our case.

Second, this case raises questions as to whether NMS is dose dependent. Studies have shown that the risk of NMS increases with higher titration rates and total doses of drug administration, other risk factors of NMS include agitation, dehydration, restraint or seclusion and elevated environmental temperature (3, 14). The patient in this case was diagnosed with NMS after taking a sustained dose of antipsychotics for 60 days, when she was admitted to the chronic ward with stable mental symptoms. Thus, other risk factors mentioned above could be excluded. Drug accumulation caused by patient is more of a chronic effect (15), rather than a short term phenomenon. The patient did not demonstrate NMS symptoms when she received risperidone 4 mg/d, and later received LAI risperidone 25 mg per 2 weeks separately after rechallenge. However, it appears as though a combination of the two induced NMS. This indicates that at least in some cases, a higher dose of antipsychotics may induce NMS, even if it is increased gradually, and the drug can be rechallenged safely at a dosage lower than that of the NMS-inducing dose, which is generally recommended after at least 2 weeks following NMS (16).

## Concluding Remarks

This case reports important information for clinicians with regard to antipsychotic drug rechallenges and proper dosing of APs to avoid or reverse NMS.

## Ethics Statement

We have obtained the written informed consent from the patient for the publication of this case study.

## Author Contributions

All authors listed have made a substantial, direct and intellectual contribution to the work, and approved it for publication.

### Conflict of Interest Statement

The authors declare that the research was conducted in the absence of any commercial or financial relationships that could be construed as a potential conflict of interest.
